# Gender differences in digital literacy: a systematic and meta-analytic review across developmental stages and socio-cultural contexts

**DOI:** 10.3389/fpsyg.2026.1673694

**Published:** 2026-02-16

**Authors:** Laure Lu Chen, Zemin Guo, Qianru Liang

**Affiliations:** 1 Department of Applied Social Sciences, The Hong Kong Polytechnic University, Kowloon, Hong Kong SAR, China; 2 Faculty of Education, The University of Hong Kong, Pokfulam, Hong Kong SAR, China; 3 Jinan University, Guangzhou, China; 4 Guangdong Institute of Smart Education, Jinan University, Guangzhou, China

**Keywords:** age, digital literacy, gender, performance-based assessment, socio-cultural context

## Abstract

Gender differences in digital literacy persist across educational stages and cultural sites, yet findings remain inconsistent. This systematic and meta-analytic review synthesizes evidence from 43 studies conducted in 31 regions between 2005 and 2023, examining gender disparities in DL performance and growth from Grade 3 to Grade 12. The cross-sectional meta-analysis, based on 67 effect sizes, revealed a consistant female advantage (*g* = +0.14, 95% *CI* [0.11, 0.18]). The longitudinal meta-analysis, incorporating six effect sizes, showed comparable growth rates for girls (*d* = 1.48, *p* < 0.001) and boys (*d* = 1.29, *p* < 0.001). Gender differences were most pronounced in upper primary and lower secondary education and unexpectedly larger in highly developed regions than in developing ones. These findings challenge traditional assumptions about gender equity and support the gender-equality paradox, suggesting that socio-cultural and developmental factors interact to shape DL outcomes. Methodological variability, including test modality and psychometric modeling, also contributed to inconsistencies across studies. This review calls for international collaboration for research and practice reforms to safeguard gender equality through education.

**Systematic review registration:**

https://osf.io/9yax4/overview.

## 1 Introduction

Digital literacy (DL) has been a sine qua non for the younger generation to thrive in the digitalized society and a crucial subset of 21st century skills (e.g., Binkley, 2012; Care and Luo, 2016), wherein information and communication technology (ICT) and artificial intelligence (AI) prevail (Carretero et al., 2017; OECD, 2015a, 2015b). Since the 1990s, DL education has gained momentum, prompting reforms in curricula, instructional practices, and policy (Law et al., 2018).

DL has been used interchangeably with terms such as ICT literacy (Ainley et al., 2012; Fraillon et al., 2014, 2018), digital competence (e.g., Hatlevik and Gudmundsdottir, 2013; Carretero et al., 2017), and computer and information literacy (CIL, e.g., Fraillon et al., 2020, 2023). Despite terminological nuances, these conceptualizations share similar fundamental elements (Voogt and Roblin, 2012). Law et al. (2018, p. 6) defined DL as “the ability to access, manage, understand, integrate, communicate, evaluate and create information safely and appropriately through digital technologies for employment, decent jobs and entrepreneurship.” Synchronizing the extant conceptualizations used by nationwide measures, DL includes the knowledge, skills, attitudes, and ethics to use various digital devices and the Internet to pursue individual goals and achieve the common good effectively (e.g., Law et al., 2022; Reichert et al., 2023).

For individual development, DL is crucial to achieve success in science, technology, engineering, and mathematics (STEM) education (Reichert et al., 2023), which, however, has been stereotyped as male-dominated (Miller et al., 2015). The stereotype of male superiority in STEM can be transmitted by peers, parents, and educators, and thus affect children’s performance, motivation, aspirations, and career choices (Baker and Jones, 1993). Girls might show a poorer STEM self-concept (Jiang et al., 2020) and display less relevant interest (Babarović et al., 2020) than boys. Children’s perceived gender gap might influence their attitudes, behavioral reactions, and decision-making (Gidengil and Stolle, 2021). In addition, parents’ stereotypes of female inferiority might lower their expectations of girls’ abilities (Tiedemann, 2000), diminish girls’ learning motivation (Sağkal and Sönmez, 2021), and impact children’s perception of gender occupational roles (Tenenbaum and Leaper, 2002). Moreover, teachers tend to overestimate boys’ abilities while underrating girls’ (Tiedemann, 2000), partly accounting for students’ career pursuits (Miller et al., 2015). These gender stereotypes could lead to a cascade effect on the societal level. Given DL’s role in STEM education and career pursuit, addressing gender equality in DL is vital for individual development, social sustainability, and the common good.

The divide in DL has been investigated for more than two decades. However, many researchers have mainly focused on how educational systems could compensate for the shortfall among the socio-economically disadvantaged group (e.g., Aesaert et al., 2015; González-Betancor et al., 2021). Gender differences in DL have been observed in many empirical studies across distinct educational systems (see reviews, Haddon et al., 2020; Siddiq and Scherer, 2019) but not well addressed in DL education, making it still challenging to launch effective interventions to ensure gender equality in academic achievement, employment opportunities, and human rights to wellbeing. Therefore, this study aims to identify the current gender gaps in DL and map out the correlates of such gaps through a systematic review and meta-analyses.

### 1.1 How do diverse measures of DL tell the gaps between boys and girls?

One correlate to the gender differences in DL could be the measures used, including self-report surveys and performance-based assessments (Haddon et al., 2020). Self-report measures assess students’ DL by asking about their attitudes, self-efficacy, interests in ICT, and/or frequency of ICT use (e.g., Almerich et al., 2021). Although Hargittai (2005) suggested that self-report measures of DL could provide a proxy for students’ DL, researchers have pointed out that self-report measures can be biased and do not accurately reflect actual DL (e.g., Aesaert and Van Braak, 2015; Haddon et al., 2020). Numerous studies provided evidence of this view, with weak congruence identified between self-report DL and actual performance (e.g., McCourt Larres et al., 2010; Mahmood, 2016). Moreover, inconsistent results were found in studies that used self-report measures to examine gender differences in DL. For instance, some studies reported that boys have higher self-efficacy of DL than girls (Cai et al., 2017; Jackson et al., 2008), whereas Rohatgi et al. (2016) found that girls had higher self-efficacy for basic tasks, whereas boys had higher ICT self-efficacy for advanced tasks.

Alternatively, performance-based assessments aim to measure students’ actual DL by allowing them to engage in specific problems, tasks, or activities to demonstrate their digital knowledge, skills, abilities, and attitudes (e.g., Aesaert et al., 2015; Jin et al., 2020; Siddiq et al., 2017). Most performance-based assessments of DL are using digital devices and consist of constrained response items (e.g., multiple-choice items; Hatlevik et al., 2017) or authentic tasks in interactive ways (ATC21S; Care et al., 2012; Chen and Rao, 2022; Fraillon et al., 2014, 2020, 2023). Therefore, more and more scholars are embracing performance-based assessments to measure students’ DL, and only studies using performance-based assessments were included in the review.

Despite the precise estimates provided by performance-based assessments, discrepancy in boy-and-girl comparisons was still observed. For example, ICILS 2013, 2018 and 2023 consistently reported that girls significantly outperformed boys in most participating countries/regions (Fraillon et al., 2014, 2020; Fraillon, 2025). In contrast, some researchers found that boys were associated with higher DL performance than girls (e.g., Calvani et al., 2012; Gnambs, 2021). Other studies indicated that boys and girls did not perform differently on DL (e.g., Claro et al., 2012; Siddiq et al., 2017). Therefore, Siddiq and Scherer (2019) reviewed the literature using performance-based assessments published between 2007 and 2017 to examine the gender gap. They reported statistically significant gender differences in favor of girls in ICT literacy performance, moderated by educational levels, with a gender gap was larger in primary schools than in secondary schools. This meta-analysis might imply that the gender gap in DL might become narrower as children grow older. However, it is still challenging to make these claims due to (1) the small effect size of the primary school sample (whose proportion is only 13.0%) and (2) the cross-sectional design of the included studies.

### 1.2 Do gender differences vary across developmental stages and socio-cultural contexts?

Stability and changes of gender differences in self-identity, attitude, and motivation have been observed in various studies (e.g., Babarović et al., 2020; Jiang et al., 2020; Sheldrake, 2018), but only a few have explored the pertaining variation in DL. Multiple-age-cohort data from Australia’s NAP–ICTL assessments show consistent female advantages in Grades 6 and 10, with varying magnitudes across cycles. Earlier cycles (2005–2011) showed larger gaps at Grade 6, while later cycles (2014–2017) showed wider gaps at Grade 10 (Ainley et al., 2007, 2010, 2012; Fraillon et al., 2015, 2018), suggesting that that socio-cultural contexts (e.g., educational policy and curriculum updates) at a specific time frame may influence DL development.

Recently, several longitudinal studies on gender differences in DL have emerged, providing useful insights into gender differences in DL trajectories across developmental stages. Lazonder et al. (2020) found no gender differences in DL growth among Dutch students in Grades 5–6. Law et al. (2022) tracked three cohorts (Grade 3, Grade 7, and Grade 9 in 2019) in Hong Kong over 2 years and found significant female advantages in growth only in the middle cohort (Grades 7 at the starting point). In contrast, Gnambs (2021) reported negligible gender differences at the age of 15 but small male advantages in DL growth at age 18 in Germany, with boys showing greater growth than girls—contradicting Law et al.’s findings. These discrepancies highlight the role of socio-cultural context in shaping DL development.

Scholars in education, psychology, and related social sciences have long examined gender differences by considering both biological and environmental factors (e.g., Baker and Jones, 1993; Falk and Hermle, 2018; Yalcinkaya and Adams, 2020). Two of the most prominent theories in this area are *gender stratification theory* and the *gender-equality paradox*. Gender stratification theory posits that individuals’ performance and competencies are shaped by anticipated opportunities, which in turn are structured by social characteristics such as gender (Baker and Jones, 1993; Guo et al., 2024). Therefore, girls raised in male-dominated societies with limited family and institutional support might be less competent than their male counterparts. In contrast, the gender-equality paradox suggests that as societies become more economically developed, priorities shift from meeting basic needs to fostering individual autonomy. This shift allows individuals to pursue personal interests and preferences, potentially amplifying innate gender-typical tendencies (Falk and Hermle, 2018; Welzel and Inglehart, 2005). Consequently, in highly developed societies, gender gaps may widen in areas like DL due to increased freedom of choice. Although the debate between these two theories remains unresolved, both highlight that gender differences are closely tied to the specific socio-cultural context. To explore this further, the present study incorporates the Gender Inequality Index (GII)—a composite measure of disparities in reproductive health, empowerment, and labor market participation (United Nations Development Programme, 2025). A higher GII indicates greater inequality and more barriers for women. By including GII, this study aims to uncover whether gender differences in DL are shaped by societal conditions, moving beyond geographic boundaries to a deeper understanding of contextual influences.

### 1.3 Research objectives and questions

Gender differences in DL and its growth remain contentious in the literature, given that individual learning might be influenced by values, stereotypical roles, norms, and educational systems that are closely connected to the culture (Gnambs, 2021; Sachdev, 2018). So far, three meta-analyses have attempted to address this issue (Siddiq and Scherer, 2019; Qazi et al., 2022; Campos and Scherer, 2024). Qazi et al. (2022) found insignificant gender differences in DL but overlooked performance-based assessment criteria and socio-cultural factors. Siddiq and Scherer (2019) found girls outperformed boys in DL, yet their broad geographic categorizations may inadequately capture nuanced socio-cultural influences, as a single continent encompasses human development disparities and socio-economic variations (United Nations Development Programme, 2025; World Bank, 2023). Campos and Scherer (2024), using ICILS 2013 and 2018 studies, also revealed a gender advantage in favor of girls. However, their focus on Grade 8 students limited the generalizability to the broader K-12 student population. Overall, although these three meta-analyses made great efforts to aggregate effect sizes to estimate gender differences in DL, they relied solely on cross-sectional studies and lacked systematic synthesis to complement the quantitative results. Their meta-analytic method is a two-level model and might overlook the potential dependencies between effect sizes extracted from some primary studies. Additionally, the moderating role of socio-cultural contexts has yet to be examined in longitudinal DL reviews, and the interactive effects of age and socio-cultural contexts remain unclear.

To our knowledge, inconsistent findings regarding gender differences in DL performance, moderated by age and socio-cultural factors, have not been studied in depth. Furthermore, existing debates on gender differences in DL fail to incorporate a developmental perspective, neglecting how socio-cultural influences may differentially shape these differences across age. To bridge these research gaps and better understand students’ DL development, the current study performs a systematic review and cross-sectional and longitudinal meta-analyses on empirical research using performance-based assessments in the past two decades (i.e., from 2003 to 2023) to investigate:

1. To what extent do variances in individual DL performances and DL growth differ in gender?
2. To what extent does age aggregate or attenuate the disparities in DL performance and DL growth between boys and girls?
3. To what extent do socio-cultural contexts, particularly geographical region and GII, contribute to the variations in DL performance and DL growth across gender and age groups?
4. What are potential study-design correlates might explain the variability of DL performance and DL growth across gender and age groups?

The present study contributes to the DL literature by addressing three unexplored dimensions: (1) conducting an indepth systematic review to uncover regional nuances in DL research on gender differences, beyond aggregate meta-analytic estimates; (2) exploring the interactions between developmental stages and region-specific DL education practices through a systematic review; and (3) integrating GII and employing longitudinal analysis of age groups alongside cross-sectional academic-age cohorts through meta-analysis to map the gender differences in specific socio-cultural contexts (see Table 1). Furthermore, the meta-analysis uses updated three-level Bayesian models to estimate gender differences in DL. These advancements—expanded geographic representativeness, dynamic socio-contextual investigation, developmental granularity, and rigorous analytic method—add novel insights into gender disparity dynamics across developmental stages in diverse cultural settings.

**Table 1:** Comparison between the current study and previous reviews on gender differences in digital literacy.

Features	Siddiq and Scherer (2019)	Qazi et al. (2022)	Campos and Scherer (2024)	Current review
Academic age range	K-12 students Primary Secondary	Students and teachers	Grade 8 students only	K-12 students Lower primary Upper primary Lower secondary Upper secondary
Socio-cultural factor	Geographic America Asia Australia Europe	Not used for meta-analysis	Geographic Asia & the Pacific Europe & North America South America	Geographic Asia Australia/the Pacific Europe North America South America Socio-economic Gender Inequality Index (GII)
Study design	Cross-sectional	Cross-sectional	Cross-sectional	Cross-sectional; Longitudinal
Digital literacy assessment	Performance-based	No constraints	Performance-based	Performance-based
Publication period	2007–2017	2006–2020	ICILS 2013 & 2018 only	2005–2023
Review method	Two-level meta-analysis	Two-level meta-analysis	Two-level meta-analysis	In-depth systematic review Three-level meta-analysis

## 2 Methods

We conducted a systematic review to compile empirical evidence addressing predefined research questions, following explicit methods to minimize bias and support evidence-based conclusions (Green et al., 2015). Our approach adapted Gough et al.’s (2012) four-step procedure: (1) formulation of research questions, (2) literature search and study selection, (3) coding study characteristics, and (4) appraisal and synthesis.

Additionally, we conducted a meta-analysis to examine the gender differences in DL and the moderating effects of age, culture, and other contextual factors. This method standardizes effect sizes across studies and enables accurate estimation of effect heterogeneity (Borenstein et al., 2010; Glass, 1976).

### 2.1 Literature search and study inclusion

#### 2.1.1 Boolean search

We conducted a Boolean search using two key terms—*digital literacy* and *gender*. We applied OR operators for interchangeable or synonymous terms and AND operators for different key terms. Specifically, the exact Boolean search was “(‘digital literacy’* OR ‘information and communication technology literac*’ OR ‘ICT literac*’ OR ‘computer and Information literac*’ OR ‘CIL literacy’ OR ‘digital competence’ OR ‘digital skills’) AND (‘gender’ OR ‘boy’ OR ‘girl’ OR ‘female’ OR ‘male’ OR ‘sex’)”.

#### 2.1.2 Search strategy

Our literature search utilized five electronic databases (i.e., EBSCO, Proquest, Scopus, Web of Science, and Wiley), with an additional manual search on international reports. We applied the search terms to the title, abstract, and keywords. We specified the publication type as peer-reviewed articles, conference proceedings, and research reports, and restricted the publication time from January 1st, 2000 to March 31st, 2023 (i.e., the date of data retrieval). Additionally, the manual search yielded nine reports: two ICILS reports from the International Association for the Evaluation of Educational Achievement (IEA), five Australian nationwide National Assessment Program—ICT literacy (NAP-ICTL) reports administered by the Australian Council for Educational Research (ACER), and two reports from the notable project entitled *Learning and Assessment for Digital Citizenship* (for short, Digital Citizenship) led by the University of Hong Kong.

#### 2.1.3 Study selection

**Figure 1:**
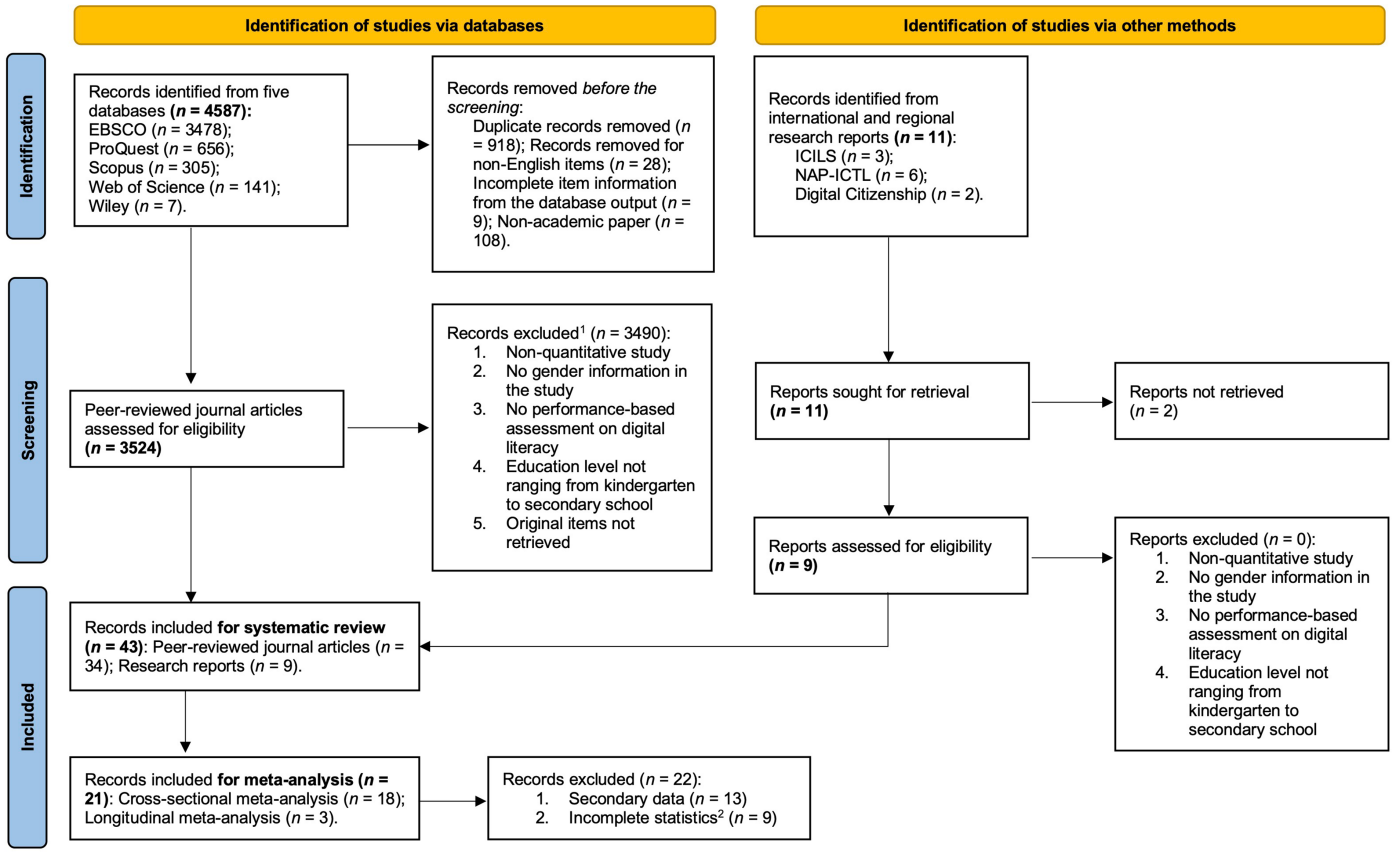
The PRISMA chart. (1) Some papers did not meet multiple criteria. (2) Nine records were excluded because neither the statistics were incomplete in the published manuscript nor the original data were retrieved from the authors.

As shown in the PRISMA chart (Figure 1, see also Page et al., 2021), the literature search returned 4,578 articles from the targeted electronic databases and 11 international and regional reports. After removing non-English items (*n* = 172) and duplications (*n* = 917), three digital literacy experts screened the remaining 3,489 articles using the following criteria: (1) English-language publication, (2) quantitative research design, (3) gender as a covariate, predictor, or group variable, (4) performance-based DL assessment, and (5) participants from kindergarten to Grade 12. When disparities occurred, the three coders discussed them until they reached a consensus. Ultimately, 34 articles and nine international reports were included for coding and analysis.

### 2.2 Coding procedure

We developed and refined the coding scheme iteratively to comprehensively extract information regarding study features (e.g., publication year, study design, data source, and sample recruitment method), participants’ characteristics (e.g., sample size, number of female and male participants, education level, and country), and DL assessment design (e.g., measurement instrument, test modality, and test fairness). Additionally, each coder reported key findings concerning the effects of gender and age to facilitate subsequent thematization and content synthesis.

### 2.3 Thematisation and content synthesis for systematic review

During the thematization and content synthesis process, we compared and contrasted data from the 43 items by identifying common themes, differences, and emerging trends. Following the coding scheme, we organized the findings into four thematic domains: study features, participant characteristics, assessment and measurement design for DL, and DL gender differences across countries/regions. These thematic domains allowed for a deeper understanding of the collected information, and the content analysis facilitated the identification of key findings and trends.

### 2.4 Statistical estimation for the meta-analysis

Three authors independently reviewed and extracted data from 18 studies. Effect sizes were computed using standardized mean differences. For cross-sectional data, we used Hedges’ *g*, which adjusts for small or unequal sample sizes (Wilson and Lipsey, 2001). For the longitudinal meta-analysis, the effect size measure was computed by subtracting the mean of the DL scores at Time 2 from the mean at Time 1 and dividing this raw mean difference by the standard deviation of the raw scores at the first time point (Morris and DeShon, 2002).

The present meta-analysis of the cross-sectional data employed a Bayesian framework due to its capacity to incorporate prior information and robustness with small sample sizes (Molto et al., 2020). Given that multiple effect sizes from the same studies, which might introduce potential dependencies among multiple effects within some primary studies, we conducted a three-level Bayesian meta-analysis (Harrer et al., 2021), using the brms package in R (Bürkner, 2017). We also performed a leave-one-out (LOO) analysis and applied Pareto-k diagnostics to identify problematic samples (Vehtari et al., 2017; Yao et al., 2017). Sensitivity analysis based on the Bayesian models was conducted using various priors and the posterior estimates were compared to evaluate the robustness of the findings.

For the longitudinal studies, it is not proper to use the multilevel meta-analytic approach due to the limited number of effect sizes (i.e., 6) from three longitudinal studies only. Therefore, a random-effects model was applied using the metafor R package (Viechtbauer, 2010), which assumes a distribution of true effect sizes and accounts for heterogeneity across studies (Borenstein et al., 2010; Hedges and Vevea, 1998). An LOO analysis with Pareto-k diagnostics was also conducted. Though exploratory, this longitudinal analysis offers preliminary insights into gender differences in DL growth.

To our best knowledge, currently, there are no proven methods developed for assessing publication bias within a three-level Bayesian meta-analytic framework. To address this, we assumed independence among effect sizes and re-estimated the model (Viechtbauer, 2010). Publication bias was then examined by using funnel plots, Egger’s test, and the Tweedie Trim and Fill methods (Borenstein et al., 2010).

## 3 Results

### 3.1 Systematic summary of the literature

#### 3.1.1 Description of selected studies

##### 3.1.1.1 Study features

A total 34 out of the 43 included studies were from journal articles (79.07%), whereas nine were from large-scale reports (20.93%). Among the 34 journal articles, most used a cross-sectional design (95.35%), whereas only two studies adopted a longitudinal design with the same participants (4.65%). The nine reports were from large-scale research projects. Two of these reports were from ICILS, primarily involving eighth-grade students in 2013 and 2018, with participation from over 20 countries (Fraillon et al., 2014, 2020). Two reports of the Digital Citizenship project adopted a longitudinal design which assessed the same three age cohorts (commencing with students at Grade 3, Grade 7, and Grade 9) from 2019 to 2021 (Law et al., 2022; Reichert et al., 2020). The other five reports were sourced from the NAP-ICTL, a nationwide study targeting Grade 6 and Grade 10 students in Australia, administered every four years from 2005 to 2017 (Ainley et al., 2007, 2010, 2012; Fraillon et al., 2015, 2018).

##### 3.1.1.2 Participant characteristics

As summarized in Supplementary Table S1, 67.44% of the studies engaged secondary students (*n* = 29), while 23.26% encompassed both primary and secondary students (*n* = 10), and the remaining four studies focused on primary-only students (9.30%). Common sampling methods included cluster (*n* = 10), stratified random (*n* = 8), and random (*n* = 6) sampling. Gender distribution was balanced (female-to-male ratio = 0.98, *SD* = 0.10). Participants’ socio-economic statuses (SES) were mainly measured by parental education/occupation, home literacy, home resources (Law et al., 2022), language integration (Hatlevik et al., 2014), school SES (Hohlfeld et al., 2013), and national composite indices (Fraillon et al., 2014, 2020).

##### 3.1.1.3 Assessment and measurement design for DL

The prevalent assessment tools used in the studied items include the CIL Scale from ICILS 2013 and 2018 (*n* = 14), the Digital Literacy Assessment from the Digital Citizenship project (*n* = 5), the ICT Literacy Scale from the NAP-ICTL project (*n* = 5). In addition to the tools used in large-scale reports, the Student Tool for Technology Literacy (ST^2^L; Hohlfeld et al., 2010, 2013), the Digital Skills Test (Gui and Argentin, 2011), and Instant Digital Competence Assessment (iDCA; Li and Ranieri, 2010; Calvani et al., 2012) were also used (both *n* = 2). Regarding the psychometric models among those primary research studies, 28 studies used item response theory (IRT) models, 14 studies measured DL based on classical test theory (CTT), and only one study adopted cognitive diagnosis models (CDM) for formative assessment purposes (Liang et al., 2021). Among the studies reporting reliability estimates (*n* = 38), the IRT-based reliability indices (*n* = 22) and Cronbach’s *α* (*n* = 14) were predominant. Notably, only 25 out of 43 (58.14%) studies further tested if gender bias existed in the measurement tools with differential item functioning, raising concerns about the validity of some gender-related findings.

##### 3.1.1.4 Geographic distribution

**Figure 2:**
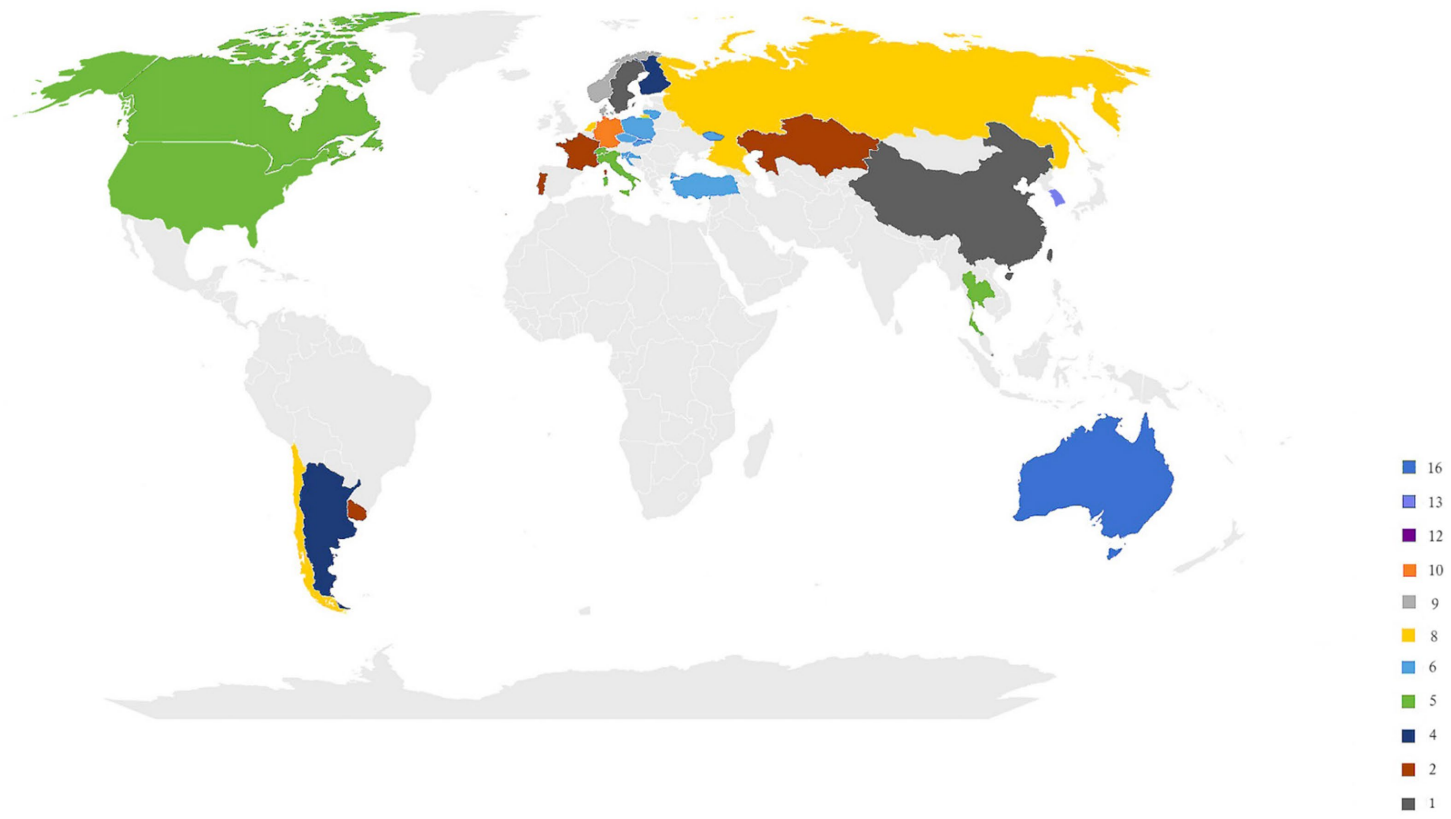
The distribution of studies investigating gender difference in DL from 2005 to 2023. The number indicates the specific number of studies investigating gender differences in digital literacy.

Figure 2 presents the distribution of studies that explored gender differences in DL between 2005 and 2023. Studies on gender differences in DL were concentrated in Europe, East Asia, and Oceania, with no relevant information from Africa, the Middle East, or Latin America. The top five regions or countries investigating the gender gap in DL are Australia (*n* = 16), South Korea (*n* = 13), Hong Kong SAR (*n* = 12), Germany (*n* = 10), Denmark (*n* = 9) and Norway (*n* = 9).

##### 3.1.1.5 Educational levels

**Figure 3:**
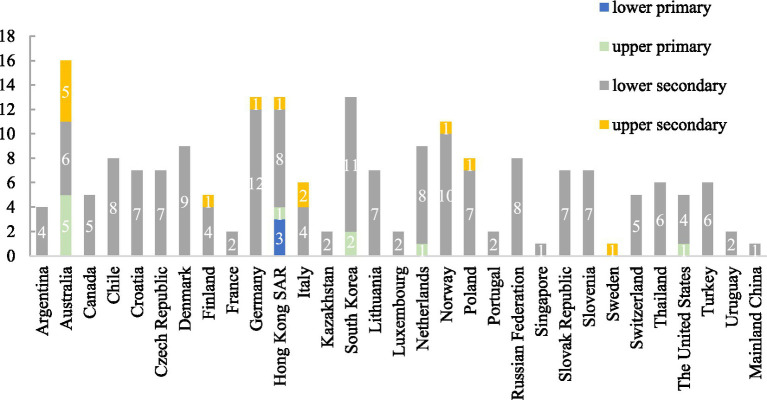
Number of studies reported gender differences in students’ DL across various countries (or regions) and educational levels from 2005 to 2023 (*n* = 39). Four studies that did not report specific gender differences were excluded from the analysis one used latent profile analysis (Heldt et al., 2020), one used gender as a control variable (Hatlevik et al., 2014), one utilized gender as a grouping variable in SEM analysis (Rohatgi et al., 2016), and one reported gender only in DIF analysis (Pan et al., 2022).

Figure 3 displays the distribution of studies reporting gender disparities in DL among participants across educational levels and countries (regions). This analysis includes only those studies that reported specific gender differences in general or specific DL (*n* = 39). The grade levels of participants were divided into four education level categories: lower primary, upper primary, lower secondary, and upper secondary school.^1^

While research in all 31 countries/regions targeted secondary students, only five countries/regions examined primary students: Australia (*n* = 5), Hong Kong SAR (*n* = 4), South Korea (*n* = 2), the Netherlands (*n* = 1), and the U. S. (*n* = 1). Notably, only Hong Kong SAR included lower primary students (*n* = 3). Lower secondary students were most frequently studied (30 of 31 regions), while upper secondary students were examined in only eight regions. Hong Kong SAR was the only region to cover all four educational levels.

#### 3.1.2 Gender differences across age cohorts and countries/regions

##### 3.1.2.1 Female advantage emerging from upper primary school across cultural contexts

**Figure 4:**
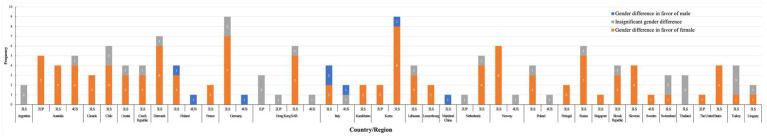
Bar plot of gender differences in DL across various countries/regions and educational levels from 2005 to 2023. 1LP = lower primary, 2UP = upper primary, 3LS = lower secondary, 4US = upper secondary.

Figure 4 summarizes gender differences in DL by participants’ age (estimated by grade level) and socio-cultural context. Studies that neither reported gender differences in DL total score (*n* = 5) nor specified differences between countries (*n* = 2) were excluded, yielding a sample of 36 studies across 31 countries/regions. No gender differences were found among lower primary students (e.g., Grade 3 students in Law et al., 2022; Reichert et al., 2020), but girls outperformed boys at upper primary levels and beyond (i.e., Grade 4 or above).

Studies targeting upper primary students reported a clear female advantage. Five Australian NAP-ICTL reports (Ainley et al., 2007, 2010, 2012; Fraillon et al., 2015, 2018)^2^ indicated that Grade 6 girls outperform boys. Similarly, two South Korean studies (Kim et al., 2014, 2021) reported that higher DL scores for girls in Grades 4 to 6, and one U. S. study (Hutchison et al., 2016) reported girls scoring higher on digital tasks at Grades 4 and 5. For secondary students, most studies—especially those using ICILS data from 2013 and 2018 (Fraillon et al., 2014, 2020)—reported girl advantage at lower secondary school levels (i.e., Grades 6/7 to Grade 9).^3^ At upper secondary school levels, several Australian NAP-ICTL reports (Ainley et al., 2010, 2012; Fraillon et al., 2015, 2018) and a Swedish study Nygren and Guath (2019) also found girls outperforming boys, particularly in corroborating digital news credibility, but no gender differences were found in sourcing and evidence.

##### 3.1.2.2 Minimal male advantage in a few developed areas

We identified six studies indicating that boys outperformed their female counterparts in DL at secondary school levels in mainland China, Finland, Germany, Italy, and South Korea, suggesting that boys’ advantages in DL likely exist in highly developed regions. Two of these six studies further elucidated the domain-specific gender differences. Among Finnish secondary students, gender differences in basic digital skills were insignificant, but males outperformed females in advanced technical and professional ICT skills (Kaarakainen et al., 2018). In Italy, boys scored higher in theoretical aspects of DL, while no significant differences were found in evaluation and operational skills (Gui and Argentin, 2011).

##### 3.1.2.3 Mixed findings within identical dataset

We spotted contradictory findings even within the same datasets (i.e., ICILS 2013/2018 data). For example, Ercikan et al. (2018) reported no significant gender differences in Argentina (Buenos Aires), Switzerland, Thailand, and Turkey using the ICILS 2013 data, whereas other studies using the same dataset reported female advantages (Alkan and Meinck, 2016; Fraillon et al., 2014). Similarly, Bokhove (2022) identified a small male advantage in South Korea using ICILS 2018 data and a two-level mixed model, contrasting with the ICILS 2018 report (Fraillon et al., 2020). These inconsistencies may stem from different analytical approaches (Hohlfeld et al., 2013).

##### 3.1.2.4 Summary

Gender differences typically emerge at the upper primary school level (aged 9 years old or so) and intensify at the secondary level. Girls generally outperform boys in performance-based tasks worldwide, whereas boys show advantages in specific DL domains, particularly in developed regions. Inconsistencies across studies—often using identical datasets—suggest influence of analytical methods and contextual factors and highlight a need to standardize effect sizes for meta-analytic estimation.

### 3.2 Meta-analytic gender variations in DL performance

Table 2 summarizes the primary studies included in the meta-analysis. For the cross-sectional meta-analysis, 18 primary studies (with extracted *k* = 67 effect sizes) published between 2005 and 2022 were included, excluding those lacking necessary statistical data. Three primary studies (with extracted *k* = 6 effect sizes) published from 2020 to 2022 were used for the longitudinal meta-analysis. Studies with multiple cohorts were split by grade level, resulting in more effect sizes than studies. The cross-sectional sample totaled 215,993 students (girls: *n* = 106,831; boys: *n* = 109,162), with sample sizes ranging from 71 to 14,485. For the longitudinal analysis, only students tracked over time were included, yielding a sample of 6,598 students (girls: *n* = 3,535; boys: *n* = 3,063), with sample sizes ranging from 71 to 5,561.

**Table 2:** Description of primary studies included in the meta-analysis.

Characteristics	*k*		Proportion of effect sizes	
	Cross-sectional	Longitudinal	Cross-sectional	Longitudinal
Sample characteristics				
*Educational level*				
Primary school	14	3	21%	50%
Secondary school	53	3	79%	50%
*Region of the study sample*				
Asia	17	3	25%	50%
Australia	11		16%	
North America	2		3%	
Europe	33	3	49%	50%
South America	4		6%	
Publication characteristics				
*Publication type*				
Peer-reviewed journal articles	16	3	24%	50%
Research reports	51	3	76%	50%
*Publication year*				
2005	2		3%	
2007	1		1%	
2008	2		3%	
2010	1		1%	
2011	2		3%	
2013	2		3%	
2014	24		36%	
2017	3		4%	
2020	23	2	34%	33%
2021	4	1	6%	17%
2022	3	3	4%	50%
Study characteristics				
*Modality of measures*				
Paper-pencil	9	3	13%	50%
Computerized	58	3	87%	50%
*Test fairness*				
Examined	54	4	81%	67%
Not examined	13	2	19%	33%

#### 3.2.1 A cross-sectional meta-analysis on gender differences

##### 3.2.1.1 Overall effect size and between-study variation

**Figure 5:**
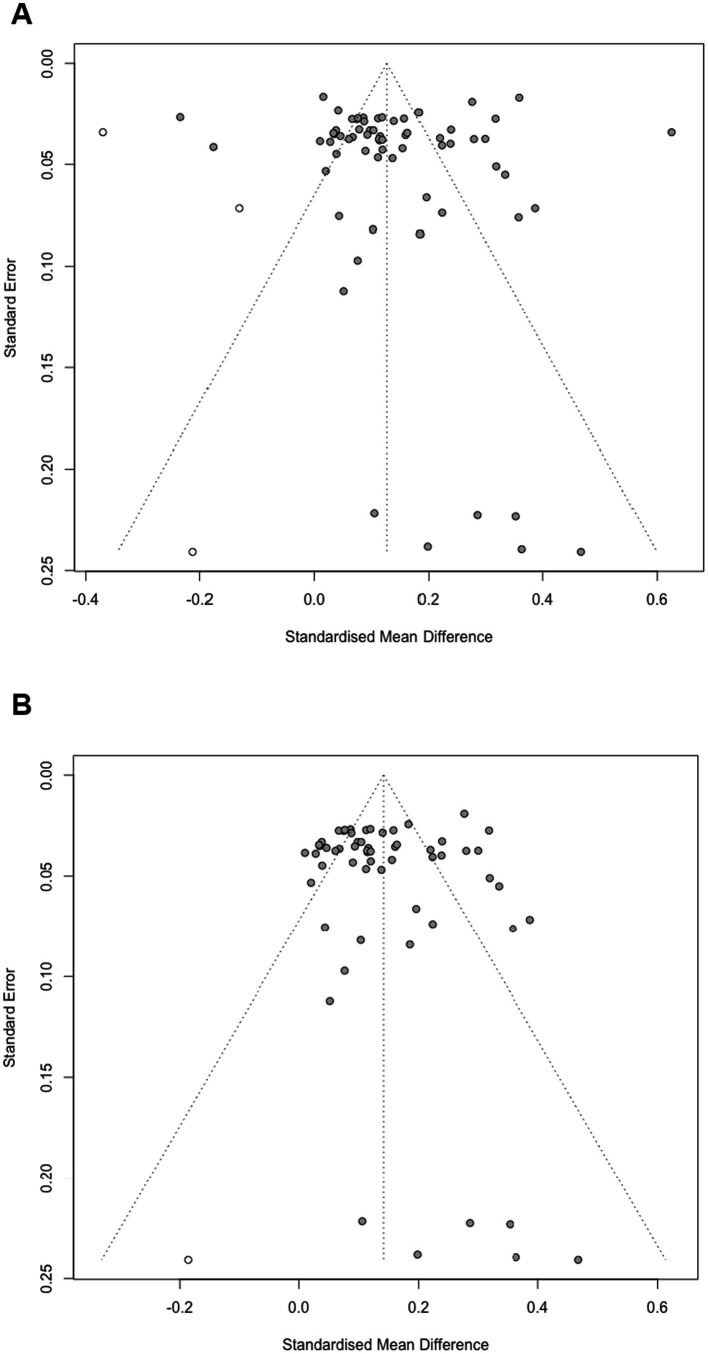
Funnel plot of the gender differences in digital literacy based on the cross-sectional studies. **(A)** Full effect sizes (*k* = 67). **(B)** Effect sizes without problematic outliers (*k* = 60).

A Bayesian random-effects meta-analysis using a normal-normal hierarchical model estimated the overall effect size and heterogeneity across 67 effect sizes. The mean effect size was 0.14 (95% *CI* [0.07, 0.21]), indicating a significant gender difference favoring girls in DL. Between-study heterogeneity (*τ*) was 0.14 (95% *CI* [0.10, 0.20]), suggesting low variability in effect sizes across studies. The relatively symmetric funnel plot of standard errors by effect size (Figure 5A) also demonstrates no publication bias in the data.

LOO analysis was used to detect whether there was any influential effect sizes on the overall effect size. The estimated expected log pointwise predictive density (elpd_loo) was −147 (*SE* = 78.2), with an effective number of parameters (p_loo) of 113.9 (*SE* = 42.0). The LOO information criterion (looic) was 294.0 (*SE* = 156.3), indicating an overfitting model with a high uncertainty. Pareto-k diagnostics were used to evaluate the reliability of the LOO estimates. Most effect sizes had Pareto-k values below 0.70, suggesting good reliability. However, seven effect sizes exceeded the critical value of 1.0 [i.e., two from Gnambs (2021), two from NAP-ICTL05 (2007), one from Kuhlemeier and Hemker (2007), one from Kim et al. (2014), and one from Hatlevik and Christophersen (2013)], indicating problematic outliers in the model. Therefore, another model was estimated based on the sample without these seven outliers, yielding a mean effect size of 0.16 (95% *CI* [0.12, 0.21]). The between-study heterogeneity (τ) of this sample was estimated at 0.08 (95% *CI* [0.05, 0.13]). Then the Pareto-k diagnostics reported that all effect sizes had values well below 1.0, suggesting acceptable reliability. In addition, based on the 60 effect sizes without outliers, a comparison of models with various priors for sensitivity examination yielded similar posterior estimates, namely, 0.16 (95% *CI* [0.12, 0.21]) and 0.16 (95% *CI* [0.12, 0.21]), suggesting that the results were robust to prior selections. Subsequent analysis was conducted using the sample of 60 effect sizes.

Next, a simple meta-analysis model was fitted to estimate the publication bias. The funnel plot (Figure 5B) was not asymmetrical around the mean effect size of the simple model (*z* = 1.78, *p* = 0.075), which indicates no strong evidence of publication bias. The limit estimate of the effect size as *SE* approached zero was *b* = 0.11 (95% *CI* [0.06, 0.15]), suggesting a small but statistically significant overall effect. The Tweedie Trim and Fill method imputed three potentially missing studies, adjusting the overall effect size from *g* = 0.14 (95% *CI* [0.10, 0.18]) to *g* = 0.13 (95% *CI* [0.10, 0.16]). These results suggest that the observed effect may be slightly overestimated due to publication bias.

**Figure 6:**
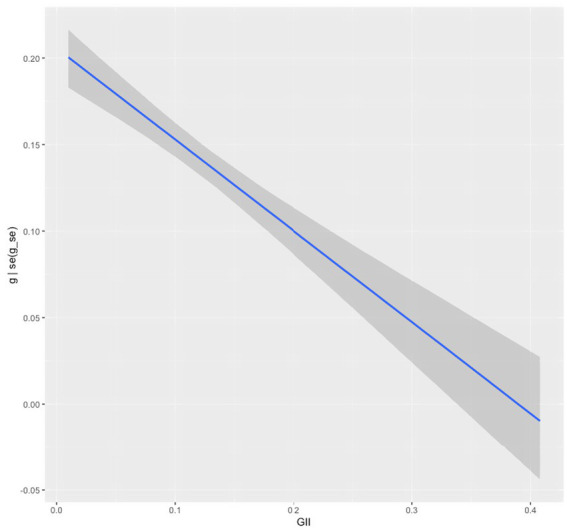
Meta-regression plot of the gender differences in digital literacy moderated by gender inequality index (GII).

Considering there are significant between-study variances in gender differences, we further investigated if the sample and study characteristics might explain the variances (Table 3). First, we used education level (also named as academic age) as a substitute for biological age to examine the age effect because most measurements of DL have been conducted within a school context and students’ age could not be explicitly identified (Kim et al., 2021). Lower secondary and upper primary had an estimated effect size of 0.05 and 0.07, respectively, both higher than the upper secondary, with 95% *CI*s not including 0, suggesting that education level was a meaningful moderator. Second, regarding the geographical region, whilst gender disparity in DL in both Europe and North America was not significantly different from that in Asia, Australia and South America had estimated effect sizes (from −0.09 to −0.06) lower than Asia, with 95% *CI*s not including 0, indicating that geographical region was a significant moderator of effect size. Third, the effect sizes were moderated by the regional GII^4^ with an estimate of −0.53 (95% *CI* [−0.64, −0.40]), indicating that areas with greater gender inequality tend to exhibit smaller gender differences favoring females in DL, whereas areas with less gender inequality are more likely to show greater female advantages in DL (see Figure 6). Fourth, the effect sizes were also moderated by the instrument fairness, with an estimate of −0.13 (95% *CI* [−0.17, −0.10]), meaning that girls’ advantage in digital literacy was smaller when measurement invariance was reported.

**Table 3:** Analysis of moderation effects by sample and study characteristics in the cross-sectional meta-analysis without outliers.

Moderator variables	*k*	*g*	*SE*	95% *CI*
Sample characteristics				
*Educational level*				
Lower Primary	1	−0.05	0.08	[−0.20, 0.10]
Upper Primary	12	0.07	0.02	[0.03, 0.10]
Lower Secondary	42	0.05	0.02	[0.02, 0.08]
Upper Secondary^1^	5	0.09	0.01	[0.06, 0.12]
*Region*				
Asia^1^	16	0.17	0.01	[0.15, 0.18]
Australia	9	−0.06	0.01	[−0.09, −0.04]
Europe	29	−0.01	0.01	[−0.03, 0.02]
North America	2	0.04	0.02	[−0.01, 0.09]
South America	4	−0.09	0.02	[−0.13, −0.05]
*Gender Inequality Index^2^*	60	−0.53	0.06	[−0.64, −0.40]
Study characteristics				
*Modality*				
Paper-pencil	7	−0.10	0.07	[−0.10, 0.19]
Computerized^1^	53	0.14	0.00	[0.13, 0.15]
*Fairness*				
Examined	50	−0.13	0.02	[−0.17, −0.10]
Not examined^1^	10	0.26	0.02	[0.23, 0.29]

^1^Reference group. ^2^Gender Inequality Index (GII): GII is a continuous, year-specific variable. Each sample’s GII was matched to the index from the study’s publication year. As GII data is unavailable for Hong Kong, the annual average for the “Very High Human Development” group was used. For Turkey, the annual average for the “Europe and Central Asia” region was applied.

#### 3.2.2 A longitudinal meta-analysis on gender differences

##### 3.2.2.1 Overall effect size and between-study variation in DL growth

**Figure 7:**
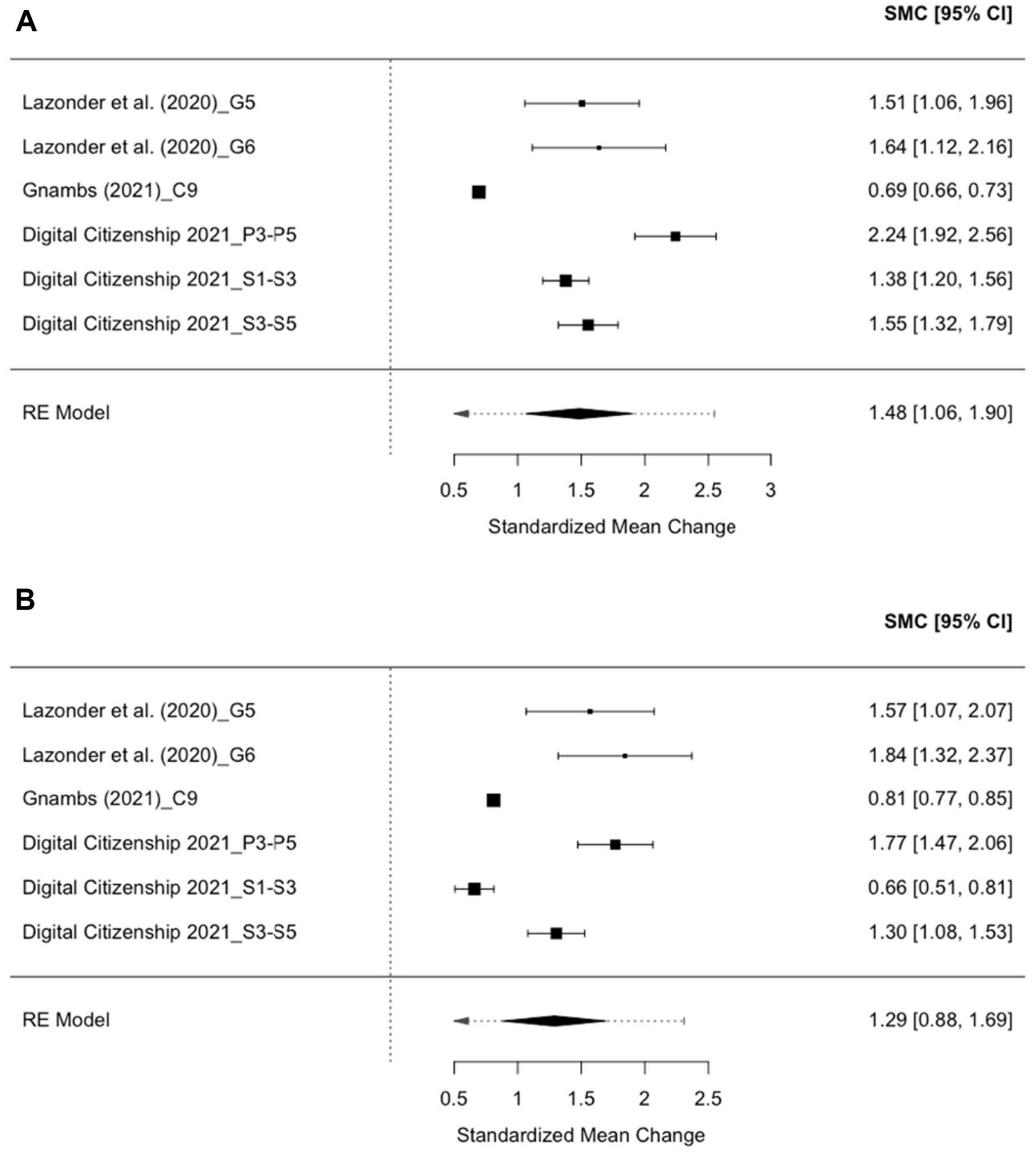
Forest plots displaying the distribution of effect sizes of DL scores based on the longitudinal studies. **(A)** DL growth among girls and **(B)** DL growth among boys.

DL Changes across genders were explored based on six effect sizes: three from primary schools and three from secondary schools. Among them, the time between the first and final tests was 2 years for five effect sizes and 3 years for one effect size. The results revealed significant growth in DL for both girls (*d* = 1.48, 95% *CI* [1.06, 1.90], *p* < 0.001) and boys (*d* = 1.29, 95% *CI* [0.88, 1.69], *p* < 0.001) among the six effect sizes (see Figure 7). Significant heterogeneity was found in the girls’ growth across studies, with *τ*^2^ = 0.25 (*SE* = 0.17). The proportion of total variability due to heterogeneity was high (*I*^2^ = 96.06%). The test for heterogeneity was significant, *Q*(5) = 206.27, *p* < 0.001, suggesting that the variation in effect sizes was not due to sampling error alone. Similarly, substantial heterogeneity was also found in the boys’ growth across studies (*τ*^2^ = 0.23, *SE* = 0.16), with a high proportion of variability due to heterogeneity (*I*^2^ = 96.39%). A significant heterogeneity test [*Q*(5) = 84.27, *p* < 0.001] indicates that the variability in effect sizes was not explained by the sampling error solely. All effect sizes across girls’ and boys’ growth models had Pareto-k values below 0.70, suggesting good reliability. Further, the comparison of estimates revealed no significant gender differences in the growth magnitude of DL between the two gender groups [*t*(6596) = 0.65, *p* = 0.52].

**Figure 8:**
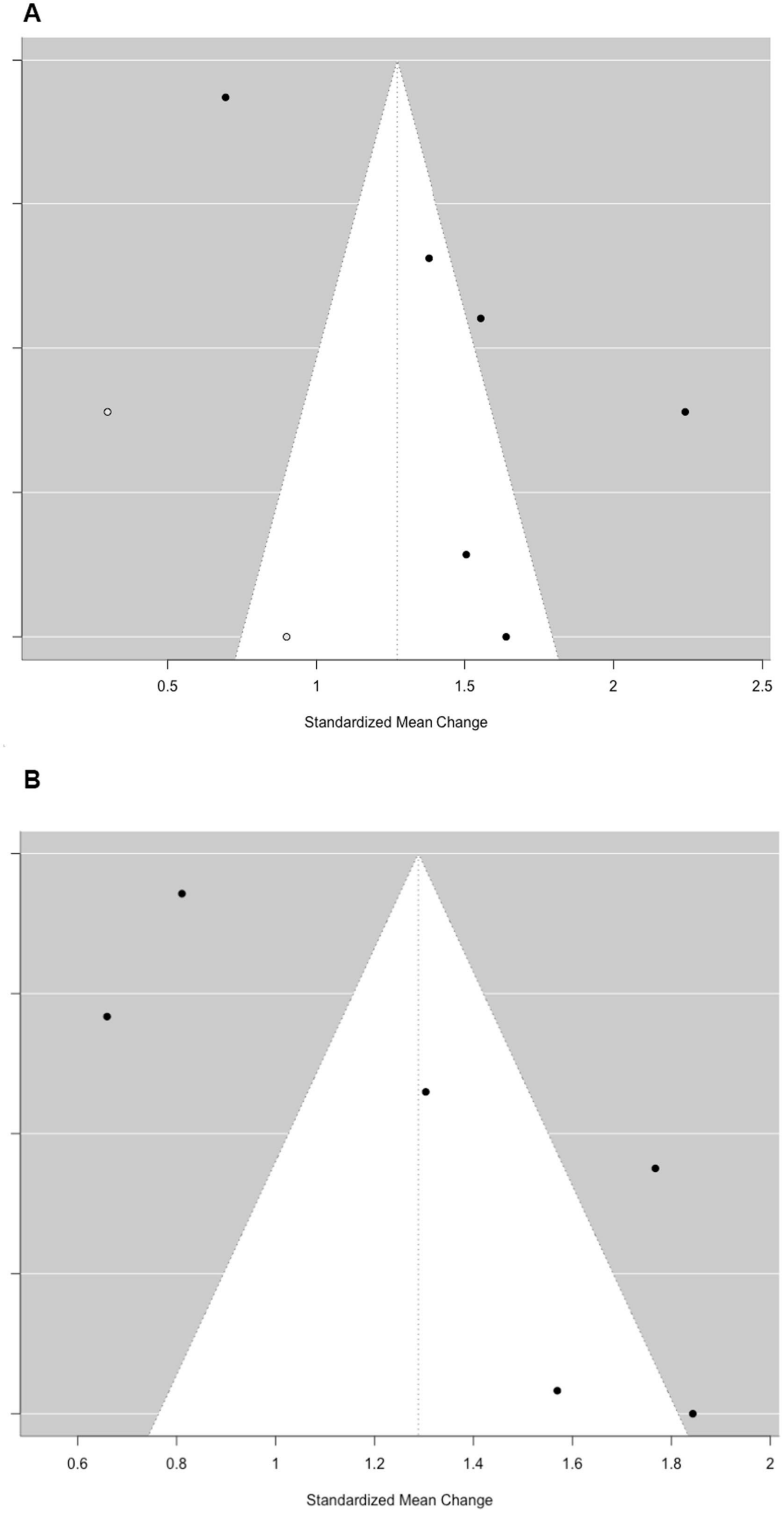
Funnel plot of the digital literacy growth across gender groups based on the longitudinal studies. **(A)** Digital literacy growth among girls. **(B)** Digital literacy growth among boys.

Then, a simple meta-analysis model of DL growth among girls and boys was fitted to estimate the publication bias, respectively. The funnel plot (Figure 8) was asymmetrical around the mean effect size of the simple models among girls (*z* = 2.09, *p* < 0.05) and boys (*z* = 2.74, *p* < 0.01), which indicates strong evidence of publication bias. The limit estimates of the effect size as *SE* approached zero were b_girls_ = 0.95 (95% *CI* [0.38, 1.51]) and b_boys_ = 0.68 (95% *CI* [0.20, 1.17]), respectively, suggesting a statistically significant overall effect for both girls and boys. The Tweedie Trim and Fill method imputed two potentially missing studies, adjusting the overall effect size among girls from d = 1.48 (95% *CI* [1.06, 1.90], *p* < 0.001) to *d* = 1.27 (95% *CI* [0.84, 1.70], *p* < 0.001), and that among boys from *d* = 1.29 (95% *CI* [0.88, 1.69], *p* < 0.001) to *d* = 1.03 (95% *CI* [0.74, 1.31], *p* < 0.001). These results suggest that the observed effects may be slightly overestimated due to publication bias.

##### 3.2.2.2 What factors might contribute to the between-study variation in DL growth between boys and girls?

Although there were no significant gender differences in the growth magnitude of DL, we investigated whether the between-study variations in mean-level change in DL might be moderated by the following six factors: region, education level, time length between assessments, GII, test modality, and test fairness. For girls, the findings reveal that only the time length between assessments moderated the between-study variations in DL growth among girls (*b* = −0.96, 95% *CI* [−1.51, −0.28], *z* = −2.74, *p* < 0.01), with *Q*_M_(1) = 7.53 (*p* < 0.001). This suggests that for each additional year of interval time, the effect size decreased by 0.96 units on average. However, region [*Q*_M_(1) = 1.43, *p* = 0.23], education level [*Q*_M_(1) = 2.89, *p* = 0.09], GII [*Q*_M_(1) = 0.39, *p* = 0.53], test modality [*Q*_M_(1) = 1.43, *p* = 0.23], and test fairness [*Q*_M_(1) = 0.06, *p* = 0.81] were not significant moderators.

For boys, only the education level moderated the between-study variations in DL growth among boys (*b* = −0.82, 95% *CI* [−1.30 to −0.35], *z* = −3.39, *p* < 0.001), with *Q*_M_(1) = 11.51 (*p* < 0.001). This finding indicates that boys’ DL growth variations were smaller in secondary schools than in primary schools. Other factors, region [*Q*_M_(1) = 0.08, *p* = 0.78], time length between assessments [*Q*_M_(1) = 1.26, *p* = 0.26], GII [*Q*_M_(1) = 0.47, *p* = 0.50], test modality [*Q*_M_(1) = 0.08, *p* = 0.78], and test fairness [*Q*_M_(1) = 1.92, *p* = 0.17], were not significant moderators.

## 4 Discussion

This systematic and meta-analytic review revealed a consistent female advantage in DL performance across primary and secondary education stages, though the extent of this advantage varies across societies. This variation suggests that the observed performance gap may reflect more than just differences in competence—it may be shaped by broader contextual influences. A context-sensitive view of students’ performance across age cohorts might help elucidate the present gender differences in DL. Diverse contextual factors, such as the cultural features of participants being assessed, individuals’ psychological and biological attributes, and research methodologies, might interactively result in the gender divide in DL. As such, we start by discussing our findings pertaining to the three guiding research questions, which prompt deeper reflection on the potential explanations for male–female differences in DL.

### 4.1 Gender differences in DL performance and cultural determinants

Convergent evidence from the systematic review and meta-analysis indicates a female advantage in DL performance, with *g* = +0.16 (95% *CI* [0.16, 0.21]), and this pattern was more evident in upper primary and lower secondary students. While no significant gender differences were found in lower primary education levels, the DL gender gap favoring girls persisted across most upper primary and secondary school students. The cross-sectional meta-analysis further supports this trend, with stronger gender effects in upper primary and lower secondary school student cohorts. Across various geographic and socio-economic regions, 28 out of 31countries/regions reported girls outperforming boys across K-12 at different ages. This female advantage may be partly explained by the individual factors: girls show higher levels of motivation and conscientiousness in low-stakes tests such as digital competence assessments, compared to their boy counterparts (e.g., Karpiński et al., 2023, using ICILS 2018 data). Similarly, Balart and Oosterveen (2019) argue that girls exhibit greater test-taking diligence, such as reviewing answers and ensuring completeness.

The systematic review revealed that most empirical studies supported girls’ advantages in DL performance, yet some non-significant gender differences were mainly found in European societies. This may reflect inconsistencies in DL education and policy in Europe. Implementing ICT education is not uniformly mandated across European countries (Jones and Procter, 2023), and some local governments faced financial and resource constraints, leading to fragmented practices and limited central oversight (Madsen et al., 2018).

Interestingly, our systematic review also indicated that boys outperformed girls in DL performance from certain high-income regions, such as Zhejiang (China)^5^, Finland, Germany, Italy, and South Korea. While the case from South Korea can be attributed to different methodologies, the remaining findings suggest socio-cultural influences. In high SES regions, where ICT access is generally equitable, students’ attitudes—such as anxiety, enjoyment, and interest—become more influential predictors of students’ DL performance (Campos and Scherer, 2024; Fraillon et al., 2015). These findings align with the gender-equality paradox, which posits that gender differences may be more pronounced in societies with high levels of human development and gender eqaulity. However, caution is warranted in generalizing this explanation based on only five cases.

Our cross-sectional meta-analysis identified the GII as a significant negative moderator of gender differences in DL, with an estimated effect size of −0.53 (95% *CI* [−0.64, −0.40]). This suggests that in more gender-equal societies, the DL gap favoring girls is wider, whereas in less equal societies—where women face greater structural barriers—the gap is narrower, though still present. This finding challenges the gender stratification hypothesis, which posits reduced gender disparities in more egalitarian contexts (Baker and Jones, 1993; Guo et al., 2024). Instead, our results align with the gender-equality paradox (Stoet and Geary, 2018; Yalcinkaya and Adams, 2020), which is often explained through post-materialist theory—the idea that economic development shifts societal values from survival needs to individual autonomy (Falk and Hermle, 2018; Welzel and Inglehart, 2005). Importantly, our findings further indicate that the paradox persists even when material and structural barriers at familial and societal levels remain, particularly in the context of DL education. As structural constraints diminish, girls’ advantages in DL become more visible. Whether the disparity favors girls or boys, the evidence indicates that socio-economic and human development foster individual expression, thereby amplifying gender differences in DL.

In sum, the persistence of gender differences in digital education underscores that humans are socialized into gender, and the enduring gender disparities in DL likely result from a combination of entrenched gender norms, structural inequalities in education and labor markets, and the politicization of gender issues. To address these challenges, educational technologists, practitioners, researchers, and policymakers must collaborate to advance the gender development agenda and eliminate the underlying risk factors.

### 4.2 The age determinant and other relevant correlates to DL disparities

Synthesizing evidence from the systematic review and cross-sectional meta-analysis, this study confirms a steady girl advantage in DL (*g* = +0.16, 95% *CI* [0.12, 0.21]). However, the influence of age on gender differences in DL appears more nuanced. Both the review and meta-analysis indicate that gender disparities begin to emerge at the upper primary level and persist through lower secondary education, with most studies reporting a female advantage. This finding regarding girl favoritism in DL also resonated with developmental research in other academic domains. For example, gender differences in STEM-related subjects were negligible throughout childhood and tend to emerge during adolescence, partially influenced by socio-cultural factors such as parental education, STEM-related abilities, beliefs, and school environments (Guo et al., 2024; Jiang et al., 2020; Lindberg et al., 2010; Wang and Degol, 2017).

The cross-sectional meta-analysis further supported this pattern, showing that girls’ advantage in DL performance at the upper primary school level (*g* = +0.07, 95% *CI* [0.03, 0.10]) and lower secondary school level (*g* = +0.05, 95% *CI* [0.02, 0.08]) was significantly different from that at the upper secondary school level (the reference group). This finding was broadly congruent with Siddiq and Scherer’s conclusions (2019), which identified a relatively larger gap at the primary school level. Although they used binary categorization of education level, the present study’s four-level categorization offers greater granularity. Nonetheless, both studies converge on the observation that gender gaps tend to narrow with age. Researchers should be cautious when interpreting the age effects of the current meta-analytic findings and develop more studies in the primary school sectors or explore biological age effects.

It should also be noted that the academic advantage of girls (Casad et al., 2017), such as DL, does not necessarily translate into increased self-efficacy or long-term success in STEM fields (Marsh et al., 2019). Although the meta-analysis found that region and test fairness were not significant moderators—suggesting the universal girls’ excellence in DL across assessment contexts—this performance edge does not always lead to equitable outcomes. Girls often need to work much harder and perform much better than boys to gain equivalent recognition, as a disparity rooted in unequal access to social capital and persistent socio-cultural biases (Guo et al., 2024; Sheldrake, 2018). Addressing this imbalance requires systematic changes. For instance, feminist pedagogy (Herman and Kirkup, 2016) should be reformed from merely promoting access to actively raising awareness of structural constraints and empowering girls to challenge professional boundaries.

Another interesting finding from the longitudinal meta-analysis revealed that DL growth was similar for boys and girls over the same time span, echoing the prior research showing stable gender gaps in STEM interest (Babarović, 2021). However, the moderation results unveiled that DL growth was smaller for secondary school-aged male students than their primary school counterparts, while the growth remained similar for girls between primary and secondary school periods. This nuance may reflect gendered developmental trajectories. For instance, Sheldrake’s national study of 11- to 14-year-olds (2018) found that males’ aspirations in STEM-related fields experienced more changes than females’, potentially affecting their performance. Nevertheless, these findings should be interpreted cautiously due to the limited number of effect sizes. Overall, the longitudinal meta-analysis is among the first to provide a developmental perspective on gender differences in DL, suggesting that future empirical studies should investigate biological, psychological, and socio-cultural factors concerning DL acquisition and instruction.

### 4.3 Methodological factors contributing to variations in the interactive effect of gender and age on DL

This study provides evidence of gender differences favoring girls in performance-based assessments of DL, despite a similar magnitude of DL growth between boys and girls across cultural sites. However, findings from the systematic review suggest that the interaction between gender and age varies across regions, indicating that methodological factors, such as assessment and analytic methods, may contribute to these variations.

Two key assessment-related factors—test modality and test fairness—appear to influence the observed age-gender interaction in DL. Most studies used computerized tests (Fraillon et al., 2014; Reichert et al., 2020), while a small proportion have used paper-and-pencil tests (Gnambs, 2021; Lazonder et al., 2020; Li and Ranieri, 2010). Interestingly, studies using paper-pencil tests in countries such as mainland China, the Netherlands, and Germany reported no gender difference or a male advantage at upper primary or secondary school levels. In contrast, studies using computerized testing conducted in the same countries and education levels typically found a female advantage (Fraillon et al., 2014; Reichert et al., 2020). These discrepancies suggest that test modality may influence the direction and magnitude of gender differences. Moreover, test fairness remains an underexamined issue. Approximately 58% of the studies in the systematic review and 39% of the studies in the meta-analysis did not report measurement invariance across gender. Meta-analytic regression further indicated that the female advantage differed between the studies that reported measurement invariance and those that did not. Because measurement invariance is essential for detecting and correcting for item bias, failure to assess its risks can conflate measurement bias with true performance differences (e.g., Hatlevik et al., 2017). Future assessment studies should establish measurement invariance—a key component of test fairness—to safeguard valid group comparisons.

Analytical choices also contribute to variations in the findings. Despite the widely acknowledged multidimensional framework of DL, most studies used unidimensional psychometric models (e.g., CTT, unidimensional IRT models) to report DL as a single construct (Aesaert and Van Braak, 2015; Claro et al., 2012; Jin et al., 2020). This simplification may obscure nuanced gender differences. For example, while Hatlevik et al. (2017) and Jin et al. (2020) found a female advantage in DL using unidimensional models, Li and Ranieri (2010) reported a reverse pattern, and Kuhlemeier and Hemker (2007) revealed insignificant gender differences. The choice of statistical model further complicates interpretation. Hohlfeld et al. (2013) demonstrated that gender differences appeared significant when using simple *t*-tests but disappeared when more complex models (e.g., multilevel modeling with covariates) were applied. Similarly, Bokhove (2022) found that Grade 8 boys outperformed girls using the ICILS 2018 dataset—contradicting the original ICILS findings—due to differences in sampling, missing data treatment, and modeling strategies.

In conclusion, methodological factors, such as assessment modality, test fairness, psychometric models, and statistical modelling, contribute to the observed interaction between gender and age in DL performance. Researchers should carefully consider these factors when interpreting and comparing findings across different studies and contexts. Future research should prioritize methodological rigor—particularly in ensuring measurement fairness and adopting multidimensional, context-sensitive models—to provide more reliable and consistent evidence on gender differences in DL performance and growth.

### 4.4 Recommendations and implications

#### 4.4.1 Research implications

Digital learning starts as early as kindergarten (Chu et al., 2024), and its effectiveness depends on the foundational digital skills of students, parents, and teachers. However, empirical studies assessing DL in young children remain scarce. Existing research, primarily from Hong Kong and Germany, has focused on Grade 3 students (aged 8–9), leaving younger cohorts underexplored. This gap is concerning, as early exposure to digital environments without adequate literacy may increase risks such as excessive screen time, anxiety, depression, and sleep disturbances (Livingstone et al., 2021; Haddon et al., 2020). Conversely, DL has shown promise in combating misinformation (Anthonysamy and Sivakumar, 2022), mitigating emotional symptoms (Livingstone et al., 2021), and protecting against cyberbullying (Tso et al., 2022). Therefore, future research should prioritize DL assessment and intervention among younger children attending kindergarten to lower primary school to foster digital savvy from an early age.

The small effect size in the present longitudinal meta-analysis limits the generalizability of gender differences in DL across educational levels. This highlights the need for more longitudinal studies that examine intraindividual and interindividual differences, incorporating biological, psychological, and socio-cultural factors. More longitudinal studies could illuminate the developmental trajectories of DL and inform curriculum designs and parenting interventions that support equitable DL development. Additionally, the paucity of DL research in Africa, the Middle East, Southeast Asia, and South America warrants future work to redress this imbalance and advantage global inclusivity and equity in digital education.

DL is a dynamic construct that must evolve alongside technological advancements and educational reforms. The IEA’s ICILS 2023 framework now includes computational thinking (CT) as a core component (Fraillon et al., 2023), and efforts are underway to validate cross-cultural assessments of ICT skills among 15-year-olds across 35 education systems. Moreover, AI literacy is increasingly recognized as part of digital competence (Reichert et al., 2023), and the updated DigComp 2.2 framework integrates AI-related content across five competence areas (Vuorikari et al., 2022). Despite these developments, standardized assessments of DL remain underdeveloped and require further scholarly attention.

Assessment modality and psychometric robustness also warrant attention. Research indicates that small-screen devices may impair reasoning performance (Sanchez and Branaghan, 2011), potentially contributing to digital divides between children using mobile devices and those with access to larger screens (Reichert et al., 2020). Yet, few studies have examined how device type influences DL outcomes. Future research should develop culturally responsive, context-sensitive, and age-appropriate DL assessments that reflect the evolving digital landscape (Chen and Rao, 2022). Additionally, some studies have not examined measurement invariance across gender before group comparison, suggesting that unbiased measurement should be further ensured for valid group comparison, and future meta-analyses could focus exclusively on studies that establish invariance.

Finally, gender-related DL studies often rely on aggregated or single scores, which may obscure nuanced differences. Evidence suggests that girls excel in communication and creativity tasks, while boys perform better in technically demanding tasks (Gebhardt et al., 2025; Haddon et al., 2020). Kaarakainen et al. (2018) reported that boys outperformed girls overall; however, gender differences were negligible for basic digital skills and were concentrated in advanced and professional technical skills. Ilmarinen and Lönnqvist (2024) advocate for disaggregating DL dimensions to better understand the gender equality paradox. Future research should investigate gender differences at the dimension levels (e.g., Liang et al., 2021), enabling gender-sensitive pedagogy and personalized instructions. Such approaches can help narrow gender gaps and support the holistic development of all learners.

#### 4.4.2 Practical implications

Despite similar growth rates in DL across genders, girls consistently outperform boys in performance-based DL assessments. Yet, females remain underrepresented in ICT-intensive fields such as computer science and AI, often due to entrenched socio-cultural stereotypes and expectations (Stoet and Geary, 2018; Iacoviello et al., 2024). This disparity highlights the need for targeted educational policies and practices to promote gender equity.

Education departments should ensure equal access to digital resources and training, while teachers should foster inclusive environments using diverse, engaging materials. Gender-integrated classrooms have shown positive academic and social outcomes (Fabes et al., 2019), and collaborative learning among students of all gender identities can further support equity. Researchers should also investigate the psychological and social mechanisms behind gender differences in DL, which could inform interventions and rigorous evaluations aimed at increasing female participation in ICT fields.

Additionally, as AI tools become embedded in K–12 education, attention must be paid to algorithmic biases stemming from underrepresented training data (Al-Alawi et al., 2021; Chandler and Munday, 2020). Educators and developers should prioritize inclusive datasets and monitor AI outputs to prevent reinforcing discriminatory patterns. These efforts are essential to ensure DL fosters equitable opportunities for all learners.

## 5 Conclusion

This study offers a comprehensive and up-to-date review of gender differences in DL performance. We enhanced our review by corroborating the systematic review with a meta-analysis of peer-reviewed journal articles and international research reports. Another key feature of this study is the usage of longitudinal meta-analysis to compare growth in DL across genders, despite its relatively small effect sizes, and the integration of socio-economic factors into the analyses. This approach provides developmental and socio-cultural perspectives on individual differences in DL over the past 5 years. Importantly, both the qualitative and quantitative findings contribute to the theories of gender stratification and gender-equality paradox, particularly in the context of digital education. Beyond advancing scholarly understanding, this study offers practical implications for policymakers and educators striving to foster gender equity in the development of knowledge, skills, and attitudes among digital citizens.

## Data Availability

The raw data supporting the conclusions of this article will be made available by the authors, without undue reservation.

## Footnotes

1 Most studies follow the 6–3-3 education system—6 years of primary, three of lower secondary, and three of upper secondary—whereas the U.S. uses a 5–4-3 model. In the 5–4-3 education system, grades 1–3 are considered lower primary, and grades 4–5 are upper primary.

2 The DL gender difference results of Ainley et al., 2007 (NAP-ICTL05) are based on Fraillon et al. (2014) (NAP-ICTL17).

3 For the educational system in the United States, the lower secondary school levels include Grade 6 to Grade 9. For the other countries/regions, the lower secondary school levels include Grade 7 to Grade 9.

4 GII data were extracted from the website of Human Development Reports of United Nations Development Programme: https://hdr.undp.org/data-center/thematic-composite-indices/gender-inequality-index#/indicies/GII.

5 As China is a social-economically diverse country, Zhejiang is one of the richest provinces (The People’s Government of Zhejiang Province, 2022). Therefore, it is considered a highly developed region.
