# Supplementary material for: Gender differences in digital literacy: a systematic and meta-analytic review across developmental stages and socio-cultural contexts
Source: Front Psychol. 2026 Feb 16;17:1673694. doi: 10.3389/fpsyg.2026.1673694 (PMC12950720; doi:10.3389/fpsyg.2026.1673694)
Supplement: Supplementary file 1 [file Table_1.DOCX]

**Table S1**

*Summary of Participants’ Characteristics of the Studies Included*

| **Author (year)** | **Country** | **Sampling method** | **Grade level or age** | **Sample size** | **Nob of Female** | **Nob of Male** | **Sample source for secondary analysis** |
| --- | --- | --- | --- | --- | --- | --- | --- |
| Ainley et al., 2007 (NAP-ICTL05) | Australia | Cluster | Grade 6 & 10 | 7393 | 3557 | 3795 | - |
| Ainley et al., 2010 (NAP-ICTL08) | Australia | Cluster | Grade 6 & 10 | 10926 | 5358 | 5480 | - |
| Ainley et al., 2012 (NAP-ICTL11) | Australia | Cluster | Grade 6 & 10 | 11023 | 5184 | 5839 | - |
| Ainley et al., 2016 | Australia, Chile, Croatia, Czech Republic, Denmark, Germany, South Korea, Lithuania, Norway, Poland, Russian Federation, Slovak Republic, Slovenia, Switzerland, Thailand, Turkey, Argentina, Canada, Hong Kong SAR, Netherlands | Cluster | Grade 8/9 | About 60000 | - | - | - |
| Alkan & Meinck, 2016 | Australia, Chile, Croatia, Czech Republic, Germany, South Korea, Lithuania, Norway, Poland, Russian Federation, Slovak Republic, Slovenia, Thailand, Turkey, Canada, Denmark, Hong Kong SAR, Netherlands, Switzerland, Argentina | - | Grade 8 | about 60000 | - | - | ICILS 2013 |
| Aydin, 2022 | Finland, South Korea | - | Grade 8 | 4725 | 2358 | 2367 | ICILS 2018 |
| Bokhove, 2022 | South Korea | Stratified random | Grade 8 | 2513 | - | - | ICILS 2018 |
| Calvani et al., 2012 | Italy | - | Grade 9 & 10 | 1056 | 457 | 599 | - |
| Davis & Dong, 2020 | Australia, Chile, Croatia, Czech Republic, Denmark, Germany, Hong Kong SAR, South Korea, Lithuania, Netherlands, Norway, Canada, Poland, Russian Federation, Slovak Republic, Slovenia, Thailand, Turkey | Cluster | Grade 8/9 | 47234 | - | - | ICILS 2013 |
| Ercikan et al., 2018 | Australia, Chile, Croatia, Czech Republic, Denmark, Germany, South Korea, Lithuania, Norway, Netherlands, Poland, Russian Federation, Slovak Republic, Slovenia, Switzerland, Thailand, Turkey, Hong Kong SAR, Argentina, Canada | Cluster | Grade 8/9 | - | - | - | ICILS 2013 |
| Fraillon et al., 2014 (ICILS 2013) | Australia, Chile, Croatia, Czech Republic, Denmark, Germany, South Korea, Lithuania, Norway, Poland, Russian Federation, Slovak Republic, Slovenia, Switzerland, Thailand, Turkey, Argentina, Canada, Hong Kong SAR, Netherlands | Cluster | Grade 8/9 | About 60,000 | 29744 | 30561 | - |
| Fraillon et al., 2015 (NAP-ICTL14) | Australia | Cluster | Grade 6 & 10 | 10562 | 5380 | 5182 | - |
| Fraillon et al., 2018 (NAP-ICTL17) | Australia | Cluster | Grade 6 & 10 | 10324 | 4923 | 5401 | - |
| Fraillon et al., 2020 (ICILS 2018) | Chile, Denmark, Finland, France, Germany, Italy, Kazakhstan, South Korea, Luxembourg, Portugal, The United States, Uruguay, Russian Federation | Cluster | Grade 8 | 46561 | 22642 | 23919 | - |
| Gnambs, 2021 | Germany | Stratified random | Grade 9 & 12 | 13943 (Time 1), 5407 (Time2) | 6927 (Time 1), 2920 (Time 2) | 7016 (Time 1), 2487 (Time 2) | - |
| Gui & Argentin, 2011 | Italy | Stratified | Grade 9 | 980 | 496 | 484 | - |
| Hatlevik & Christophersen, 2013 | Norway | - | Grade 9 | 4087 | 2023 | 2064 | - |
| Hatlevik et al., 2014 | Norway | Convenience (e.g., volunteers) | Grade 7 | 1793 | 895 | 898 | - |
| Hatlevik et al., 2017 | Norway | Random | Aged 15-16 | 919 | 468 | 451 | - |
| Hatlevik et al., 2018 | Australia, Chile, Croatia, Czech Republic, Denmark, Germany, South Korea, Lithuania, Norway, Poland, Russian Federation, Slovak Republic, Slovenia, Turkey | Stratified | Grade 8/9 | 45910 | - | - | ICILS 2013 |
| Heldt et al., 2020 | Czech Republic, Denmark, Germany | - | Grade 8 | 3238 | 1640 | 1598 | ICILS 2013 |
| Hohlfeld et al., 2013 | The United States | Stratified | Grade 8 | 1513 | 766 | 747 | - |
| Hutchison et al., 2016 | The United States | Purposeful | Grade 4 & 5 | 1262 | 648 | 614 | - |
| Ihme et al., 2017 | Germany, Denmark, Norway, Netherlands, Switzerland | - | Grade 8/9 | 11850 | 6060 | 5789 | ICILS 2013 |
| Jin et al., 2020 | Hong Kong SAR | - | Grade 3, 7, & 9 | 1989 | 1014 | 953 | Digital Citizenship 2020 |
| Kaarakainen et al., 2018 | Finland | - | Aged 12-22 | 3159 | 1529 | 1630 | - |
| Karpiński et al., 2023 | Denmark, Russian Federation, South Korea, Finland, The United States, Germany, Portugal, France, Luxembourg, Chile, Italy, Uruguay, Kazakhstan | - | Grade 8 | 40403 | - | - | ICILS 2013 |
| Kim et al., 2014 | South Korea | Stratified random | Grade 4-6 | 11767 | 5530 | 6237 | - |
| Kim et al., 2021 | South Korea | Stratified random | Grade 4-9 | 25048 | 12402 | 12646 | - |
| Kuhlemeier & Hemker, 2007 | Netherlands | Stratified random | Grade 7 & 9 | 2345 | 1126 | 1219 | - |
| Law et al., 2022  (Digital Citizenship 2022) | Hong Kong SAR | Stratified random | Grade 5, 9 & 11 | 1971 | - | - | - |
| Lazonder et al., 2020 | Netherland | - | Grade 5 & 6 | 151 | 71 | 80 | - |
| Li & Ranieri, 2010 | Mainland China | Random | Grade 9 | 317 | 149 | 168 | - |
| Liang et al., 2021 | Hong Kong SAR | Random | Grade 3 | 642 | 307 | 335 | - |
| Majid et al., 2020 | Singapore | Random | Grade 9 | 3306 | 1431 | 1875 | - |
| Nygren & Guath, 2019 | Sweden | Convenience (e.g., volunteers) | Aged 16-19 | 448 | 269 | 179 | - |
| Pagani et al., 2016 | Italy | Stratified random | Grade 10 | 1466 | 777 | 689 | (1) Previous empirical study conducted by the same author; (2) Italian national institute for the evaluation of the school system (INVALSI) |
| Pan et al., 2022 | Hong Kong SAR | - | Grade 5, 9, & 11 | 1968 | - | - | Digital Citizenship 2020 |
| Punter et al., 2017 | Czech Republic, Poland, Norway, Netherlands, Germany, Slovak Republic, Croatia, Slovenia, Lithuania | Random | Grade 8/9 | 25133 | 12320 | 12795 | ICILS 2013 |
| Reichert et al., 2020 (Digital Citizenship 2020) | Hong Kong SAR | Stratified random | Grade 3, 7 & 9 | 2046 | 1014 | 953 | - |
| Ritzhaupt et al., 2013 | The United States | - | Grade 6, 7 & 8 | 5990 | 2995 | 2995 | - |
| Rohatgi et al., 2016 | Norway | - | Grade 9 | 2436 | 1223 | 1213 | ICILS 2013 |
| Tomczyk & Eger, 2020 | Poland | Random | Aged 15-21 | 1693 | 848 | 845 | - |

*Notes*. - Not mentioned/measured or not applicable in the study; Nob = Number of observations; South Korea refers to the Republic of Korea.
